# Evaluating tendon transfers in irreparable rotator cuff tears: A systematic review of clinical outcomes and failure rates

**DOI:** 10.1177/17585732251368884

**Published:** 2025-08-14

**Authors:** Marc Daniel Bouchard, Nikhil Aman Patel, Carl Keogh, Danielle Dagher, Brooke Mackinnon, Sophia Argyropoulos, David Slawaska-Eng, Moin Khan, Bashar Alolabi

**Affiliations:** 1 Division of Orthopaedic Surgery, 3710 McMaster University, Hamilton, ON, Canada; 2 School of Medicine and Health Sciences, 40323 Royal College of Surgeons in Ireland, Dublin, Ireland; 3 57978 Beaumont Hospital, Dublin, Ireland; 4 Department of Health Research Methods, Evidence and Impact, 152642 McMaster University, Hamilton, ON, Canada; 5 Division of Orthopaedic Surgery, 3710 St.Joseph's Hospital, Hamilton, ON, Canada; 6 Division of Orthopaedic Surgery, 40285 Oakville Trafalgar Memorial Hospital, Oakville, ON, Canada

**Keywords:** tendon transfer, rotator cuff injury, irreparable rotator cuff tear, transfer failure, shoulder surgery, patient-reported outcome measures

## Abstract

**Background:**

Irreparable rotator cuff tears (IRCTs) pose a clinical challenge, particularly in younger patients. Tendon transfers (TTs) have emerged as a joint-preserving surgical option. However, the clinical outcomes, failure rates, and complication profiles associated with different TTs remain incompletely defined. This systematic review evaluates outcomes across various TT techniques used to manage IRCTs.

**Methods:**

A systematic search of MEDLINE, Embase, and Emcare databases was conducted for studies involving adults with diagnosed IRCTs reporting patient-reported outcomes and failure rates following TT. Non-English studies, conference abstracts, case reports, and studies with <12 months follow-up were excluded. Descriptive summaries and pooled analyses were performed by tendon type.

**Results:**

Thirty studies (980 patients, 994 shoulders) were included. The mean patient age was 58.9 years, with a mean follow-up of 44.7 months. Latissimus dorsi (LD) transfers were most commonly reported. All tendon types showed significant improvements in patient-reported outcomes. Teres major transfers exhibited the highest Constant-Murley Score improvement (+40) and greatest pain reduction (VAS-5.6), although based on a single study. Failure rates ranged from 4.2% to 14.1%.

**Conclusions:**

TTs improve pain and function in IRCTs. LD transfers remain the most widely used. Variability in techniques and outcomes highlights the need for standardized protocols and further high-quality research.

## Introduction

Shoulder injuries are among the most common musculoskeletal conditions in adults, often resulting from chronic, repetitive, overhead motions.^1^ The primary causes of shoulder pain include rotator cuff disorders, glenohumeral joint pathology, and acromioclavicular joint disease.^2^

Rotator cuff tears (RCTs) are a leading cause of shoulder pain, with age-related degeneration and inflammation significantly contributing to their high incidence, particularly in the supraspinatus tendon.^3–5^ While acute traumatic tears are less common, they are more frequently observed in younger individuals.^6^ Surgical intervention is generally reserved for patients with acute tears or persistent pain and functional limitations that fail to improve with conservative measures.^5^

Irreparable rotator cuff tears (IRCTs) represent a clinically challenging subset of RCTs. These tears vary in clinical presentation and are not always correlated with imaging findings, making their management complex.^7,8^ Definitions of IRCTs in the literature differ, with some studies categorizing them as tears ≥5 cm in diameter, those involving multiple tendons, or cases with Goutallier grade 3 fatty infiltration, often accompanied by significant muscle atrophy.^7–9^ Intraoperative assessment frequently serves as the definitive diagnostic tool, guiding subsequent treatment decisions.^8^

IRCTs predominantly affect older individuals due to age-related degeneration and reduced healing potential; however, younger patients may also experience traumatic IRCTs.^10^ Regardless of age, these tears can lead to chronic pain, functional impairments, and diminished quality of life.^10^ Surgical options for IRCTs range from graft interposition and bridging reconstructions to tendon transfers (TTs) and reverse total shoulder arthroplasty (RTSA). The choice of intervention often depends on patient-specific factors, including age, activity level, and the presence of degenerative changes.^11^ TTs, in particular, are joint-preserving procedures that involve transferring tendons from other anatomical locations to restore shoulder stability and dynamic function.^10,12,13^

The type of TT selected is generally determined by the tear's location and the associated biomechanical demands. Posterosuperior IRCTs are typically treated with latissimus dorsi (LD) or lower trapezius (LT) TTs, whereas anterosuperior IRCTs often involve the pectoralis major (PM) transfer.^10,14^ Middle trapezius (MT) transfers, while less common, are primarily used for isolated irreparable supraspinatus tears and may not be applicable to massive RCTs.^11,14^ Rehabilitation following TTs generally spans 6–12 months and is associated with significant improvements in patient-reported pain and functional outcomes, although the absence of standardized treatment protocols often results in variability in clinical outcomes.^10^

While prior literature has demonstrated that TTs are a successful method for managing IRCTs, this systematic review evaluated the clinical outcomes, failure rates, and complications associated with various types of TTs. Given the variability in tear patterns, surgical techniques, and rehabilitation protocols, the goal was to broadly summarize available TT options for IRCTs rather than directly compare procedures intended for identical indications. By synthesizing the current evidence, this review provides clinicians with valuable insights to support surgical decision-making and optimize patient care.

## Methods

This systematic review was reported in accordance with the Preferred Reporting Items for Systematic Reviews and Meta-Analyses (PRISMA) guidelines.^15^

### Search strategy and criteria

A comprehensive search strategy was developed for the EMBASE, OVID Medline, and Emcare databases. Manual searches were performed to ensure the inclusion of all relevant literature by reviewing reference lists of pertinent articles from database inception until August 2024. The search utilized Medical Subject Headings (MeSH) terms: (“rotator cuff injury” OR “rotator cuff tear” OR “rotator cuff arthropathy”) AND (“irreparable”) AND (“tendon transfer” OR “latissimus dorsi tendon transfer” OR “lat tendon transfer” OR “trapezius tendon transfer” OR “rotator cuff tendon transfer”). The Boolean operators are indicated in capital letters (Supplemental Table i). Two investigators (MDB, NAP) independently screened the titles, abstracts, and full-text articles resulting from the searches. Disagreements were resolved through discussion with a senior author (MK). Additionally, all references cited in the identified reviews were manually searched for additional articles not captured in the initial search.

### Eligibility criteria

Studies were eligible for inclusion if they involved adults aged 18 years or older with an established diagnosis of an IRCT (population) and reported on both patient-reported outcomes and failure rates following TT surgery. Failure was defined variably across studies, including imaging-confirmed re-tears, revision surgery, or conversion to RTSA. Due to inconsistent definitions and reporting, subgroup analysis by failure type was not feasible. There were no restrictions on the year of publication. All non-English studies, conference abstracts, case reports, and case series were excluded. Studies were also excluded if patients had potentially confounding concomitant shoulder procedures (i.e. arthroscopic or open rotator cuff repair) and a post-operative follow-up period less than 12 months. However, studies permitting partial repair in the setting of irreparable tears were included if TT was the primary intervention under investigation. Additionally, studies without an available full-text article were excluded.

Across included studies, criteria for TT typically involved symptomatic IRCTs with significant pain and/or weakness refractory to conservative management. Imaging findings frequently cited included RCTs ≥5 cm, Goutallier grade ≥3 fatty infiltration, Patte stage 3 tendon retraction, and/or an acromiohumeral interval (AHI) < 7 mm. Patients were typically offered TT after failure of at least 6 months of physiotherapy. Age thresholds for considering rTSA versus TT were variably reported, with TT generally favored in patients younger than 65 years.

### Assessment of study quality

The methodological index for non-randomized studies (MINORS) score was used to assess the quality of studies included in the review with a maximum score of 16 for non-comparative studies and 24 for comparative studies.^16^ For this review, a score of ≤8 was considered to be of poor quality, 9–14 moderate quality, and 15–16 good quality for non-comparative studies. These scores were ≤14, 15–22, and 23–24 for comparative studies, respectively (Supplemental Table ii). All included studies were assessed by two reviewers (MDB, NAP) who independently completed the quality assessments; a third reviewer (MK) helped resolve any disagreement.

### Data collection and abstraction

Two reviewers (MDB, NAP) extracted data from eligible studies using a standardized data collection form on Microsoft Excel (version 16.90). The information collected included study characteristics (author, year of publication, level of evidence, study design, sample size, length of follow-up), patient demographics (age, sex), clinical variables (tear location, tendon transferred, surgical technique, rehabilitation protocol), and outcomes (patient-reported outcome measures, recurrence/failure rates).

### Statistical analysis

All results were summarized descriptively using means and proportions. Data were pooled by tendon type and presented as weighted means along with range or standard deviation (SD) when appropriate. Agreement between investigators was assessed with a kappa coefficient. A kappa statistic is a measure of inter-observer variation. A grading system was used for the kappa coefficient whereby a kappa: 0.00–0.21 is considered slight agreement; 0.21–0.40 is considered fair agreement; 0.41–0.60 is considered moderate agreement; 0.61–0.80 is considered substantial agreement; and 0.81–1.00 is considered almost perfect agreement.^17^ Any disagreements were resolved through discussion with a third investigator and senior author. Data were analyzed using Microsoft Excel (version 16.90).

## Results

### Search results

A total of 862 articles were identified from the search of the EMBASE, OVID Medline, and Emcare databases (Supplemental Table i), and an additional four citations were considered for inclusion from a manual search systematic reviews on similar topics. Following duplicate removal, 470 studies were screened by title and abstract. After screening by title and abstract, 92 studies remained for full-text screening, of which 30 were ultimately included in this review (Figure 1). Agreement between the reviewers was substantial at the title and abstract stage (*κ* = 0.68) and almost perfect at the full-text stage (*κ* = 0.93).

**Figure 1.**
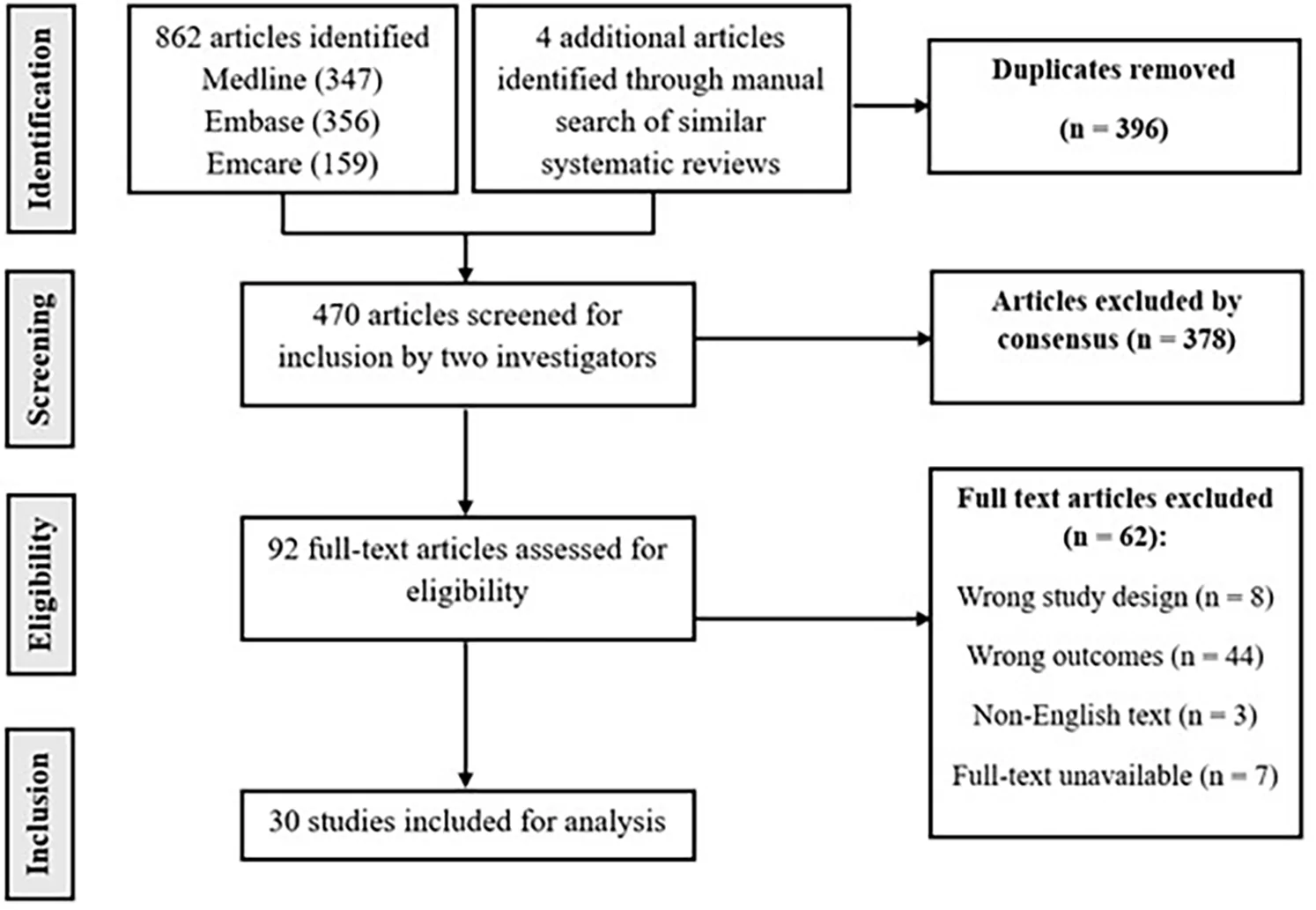
Preferred Reporting Items for Systematic Reviews and Meta-Analyses (PRISMA) diagram detailing the identification, screening, eligibility, and inclusion of studies.

The review included 18 non-comparative studies and 12 comparative studies. MINORS scores for non-comparative studies ranged from four to 13, with the majority graded as moderate quality according to the standards laid out. Three studies were graded as poor quality (≤8/16). This was primarily due to a lack of unbiased study endpoints and insufficient sample size calculations. The scores for the 12 comparative studies ranged from 16 to 21, with all but two studies graded between 18 and 20 out of the possible 24. No studies were rated as good (23-24/24) or poor quality (≤14/24) by these same standards (Supplemental Table i).

### Study characteristics and patient demographics

Table 1 summarizes the characteristics of the included studies and patient demographics. This review consisted of 30 studies, encompassing a total of 980 patients (620 males and 342 females; sex not reported in one study (*n* = 18))^18^ and 994 shoulder repairs. The mean age of patients at the time of surgery was 58.9 years (range 49–67), with a mean follow-up duration of 44.7 months (range 13–112) across all studies. The included studies were primarily retrospective cohort studies (*n* = 24) while the remainder were prospective cohort studies (*n* = 6). Individual cohort sizes ranged from 11 to 93 shoulders.

**Table 1.** Study characteristics and patient demographics.

Author (year)	Study design	MINORS score	Sample size	Mean age, years (range)	Sex (% female)	Mean follow-up time, months (range)	Tendon type
Gerhardt (2010)^18^	RCS	9	18	55.8 (49.8–61.8)	NR	70.8 (60–87)	Combined LD & TM
Baek (2022)^19^	RCS	16	30	64.1 (57.9–70.3)	43.3	38.1 (24.4–51.8)	Combined LD & TM
Boileau (2018)^20^	RCS	10	26	64.5 (29–83)	46.2	52.0 (24–104)	Combined LD & TM
Elhassan (2020)^21^	RCS	8	56	53.0 (23–79)	30	13.0 (7–51)	LD
Suh (2019)^22^	RCS	9	23	57.5 (53.1–61.9)	13	56.4 (8.4–104.4)	LD
Grimberg (2015)^23^	PCS	12	55	62.0 (31–75)	54.5	29.4 (24–42)	LD
Warner (2001)^24^	RCS	9	22	57.6 (18–78)	32	27.9 (12–75)	LD
El-Azab (2015)^25^	RCS	11	86 (93 shoulders)	56.0 (40–72)	25.8	111.6 (79.2–140.4)	LD
Aoki (1996)^26^	PCS	9	10 (12 shoulders)	64.0 (48–82)	8.3	35.6 (26–42)	LD
Nové-Josserand (2008)^27^	RCS	6	27	55.5 (36–71)	48	32.0 (20–44)	LD
Clavert (2020)^28^	RCS	11	14	52.0 (46–63)	29	57.4 (*NR*)	LD
Hanson (2023)^12^	PCS	19	17	49.0 (35–57)	71	84.0 (60–126)	LD
Valenti (2019)^29^	PCS	18	31	59.2 (37–66)	49	22.0 (12–50)	LD
Ersen (2014)^30^	RCS	11	11	54.6 (47–65)	63.6	38.0 (12–62)	LD
Iannotti (2006)^31^	RCS	11	14	54.8 (44–68)	35.7	39.0 (24–89)	LD
Paribelli (2015)^32^	PCS	20	20	62.5 (45 -77)	35	33.6 (24–60)	LD
Zafra (2009)^33^	RCS	4	18	54.0 (37–54)	72.2	28.0 (12–58)	LD
Baek (2022)^34^	RCS	10	LD: 48 LT: 42	LD: 60.9 (56.4–65.4) LT: 63.3 (57.5–69.1)	LD: 33.3 LT: 35.7	24.0 *(NR)*	LD; LT
Kany (2024)^35^	RCS	20	62	62.1 (42.7–81.4)	45	32.0 (24–62)	LD
Kany (2023)^36^	RCS	21	62	67.4 (50.2–80.7)	40	38.5 (24–66)	LD
Celli (2023)^37^	RCS	9	24	59.0 (43–65)	50	125 (120–144)	TM
Kim (2024)^38^	RCS	19	26	63.5 (56.2–70.8)	38.5	37.6 (27–54)	MT
Marigi (2023)^13^	RCS	19	72	56.8 (48.9–64.7)	19.4	37.2 (20.4–54)	LT
Baek (2023)^39^	RCS	13	22	63.3 (51–74)	36.4	28.9 (24–44)	MT
Lederer (2011)^40^	RCS	18	53 (54 shoulders)	63.0 (48–81)	24.1	35.0 (12–78)	PM
Gavriilidis (2009)^41^	RCS	10	15	61.9 (55.3–68.5)	33.3	37.0 *(NR)*	PM
Elhassan (2008)^42^	RCS	18	11	58 (45–77)	36.4	53.0 (25–82)	PM
Ernstbrunner (2019)^43^	RCS	11	22 (24 shoulders)	52.4 (35–67)	8.3	19.7 (18–22)	PM
Jost (2003)^44^	RCS	20	28 (30 shoulders)	53.0 (35–67)	6.7	32.0 (24–70)	PM
Valenti (2014)^45^	PCS	19	15	57.0 (37–66)	33.3	24.0 (12–50)	PM

NR: not reported; RCS: retrospective cohort study; PCS: prospective cohort study; MINORS: methodological index for non-randomized studies; LD: latissimus dorsi; LT: lower trapezius; TM: teres major; MT: middle trapezius; PM: pectoralis major.

Table 2 provides pooled patient demographics categorized by tendon type and their respective indications for transfer. The LD tendon was the most frequently evaluated tendon type, utilized in 17 studies,^21–36^ all for posterosuperior IRCTs. PM transfers were evaluated in six studies^40–45^ for either anterosuperior and/or subscapularis IRCT. Note that in four of the studies,^40–42,45^ the PM tendon was transferred over the coracoid/conjoined tendon while the other two studies^43,44^ passed the tendon under these structures. The lower and middle trapezius tendons were utilized in two studies each, but with different indications. The LT transfers were done for posterosuperior IRCTs^13,34^ while the MT transfers were done for isolated irreparable supraspinatus tears (IISSTs).^38,39^ A teres major (TM) transfer was done in only one study,^37^ with a long follow-up time of 125 months (range 120–144), while a combined LD and TM transfer was done in three studies,^20–22^ all for posterosuperior IRCTs. The study by Baek (2022)^34^ reported both LD and LT TTs, leading to a total of 31 populations despite the inclusion of only 30 studies (Tables 1 and 2). The mean age of patients varied slightly by tendon type, with the PM group exhibiting the lowest mean age at 58.2 years (range 52–63), while the MT group had the highest mean age at 63.4 years (range 51–74) (Table 2).

**Table 2.** Patient demographics pooled by tendon type and indication for transfer.

Tendon type	Indication	Number of studies	Sample size	Mean age (range), years	Sex % female (range)	Mean follow-up time (range), months
Latissimus Dorsi	Posterosuperior IRCT	17	576 (585 shoulders)	58.8 (49–67)	38.8 (8–72)	45.3 (13–112)
Teres Major	Posterosuperior IRCT	1	24	59.0 (43–65)	50.0 (NA)	125 (120–144)
Lower Trapezius	Posterosuperior IRCT	2	114	59.2 (49–69)	25.4 (19–36)	32.3 (20–54)
Middle Trapezius	Isolated irreparable supraspinatus tears	2	48	63.4 (51–74)	37.5 (36–39)	33.6 (24–54)
Pectoralis Major	Anterosuperior and/or subscapularis IRCT	6	144 (149 shoulders)	58.2 (52–63)	20.8 (7–36)	34.7 (20–53)
Combined (LD & TM)	Posterosuperior IRCT	3	74	62.2 (56–64)	44.7 (43–46)	50.9 (38–71)

NA: not applicable.

### Patient-reported outcome measures

Table 3 summarizes the changes in patient-reported outcome measures (PROMs) from baseline to the last follow-up where all outcomes are presented as pooled weighted means (SD). The data from the TM group represents only one study^37^ and is therefore not pooled. The same applies to the Visual Analog Scale (VAS), American Shoulder and Elbow Surgeons (ASES), and University of California-Los Angeles (UCLA) scores from the combined LD & TM row.^19^

**Table 3.** Patient-reported outcome measures (PROMs) change from baseline to last follow-up.

Tendon type	CMS	VAS pain	ASES	UCLA
Latissimus Dorsi	28.1 (2.4)	−4.4 (0.7)	33.8 (11.6)	18.7 (1.9)
Teres Major	40	−5.6	NR	NR
Lower Trapezius	NR	−4.2 (1.6)	26.9	NR
Middle Trapezius	18.2 (0.6)	−2.6 (0.3)	27.9 (0.2)	7.2 (0.9)
Pectoralis Major	25.0 (2.9)	NR	NR	NR
Combined (LD & TM)	20.9 (2.0)	−3.5	34.3	8.7

All outcomes are presented as weighted mean (SD).

CMS: Constant-Murley Shoulder Score (0–100 with higher score being better); VAS: Visual Analog Scale for pain (0–10 with lower score being better); ASES: American Shoulder and Elbow Surgeons Score (0–100 with higher score being better); UCLA: University of California-Los Angeles Shoulder Scale (0–35 with higher score being better); NR: not reported.

The LD tendon showed substantial gains in the Constant-Murley Shoulder (CMS) score, with a weighted mean improvement of 28.1 (SD 2.4). Pain reduction, as measured by the VAS, was also substantial for LD transfers, with a mean decrease of 4.4 (SD 0.7) while increases in ASES and UCLA scores for LD were 33.8 (SD 11.6) and 18.7 (SD 1.9), respectively.

The TM tendon yielded the highest CMS improvement, with a mean change of 40 points, and reported the largest reduction in VAS pain at 5.6. However, ASES and UCLA scores were not reported for TM transfers.

LT transfers showed a pain reduction of 4.2 (SD 1.6) on the VAS scale and an ASES improvement of 26.9. CMS and UCLA scores were not reported for this tendon type. MT transfers exhibited a smaller mean improvement in CMS of 18.2 (SD 0.6), with a VAS pain reduction of 2.6 (SD 0.3). The ASES score improvement was 27.9 (SD 0.2), while the UCLA score showed the lowest increase among all groups, with a mean of 7.2 (SD 0.9).

PM transfers resulted in a CMS improvement of 25.0 (SD 2.9). However, data for VAS, ASES, and UCLA scores were not reported.

For combined LD & TM transfers, the CMS score improved by 20.9 (SD 2.0), while VAS pain decreased by 3.5. ASES and UCLA scores increased by 34.3 and 8.7, respectively (Table 3).

### Failure rates of tendon transfers

Table 4 summarizes the reported failure rates for TTs categorized by tendon type. Failure was defined variably across studies. In this review, failure was defined as either a documented re-tear of the transferred tendon on imaging (magnetic resonance imaging or ultrasound) or the need for conversion to RTSA.

**Table 4.** Tendon transfer failure rates by tendon type.

Tendon type	Failure rate (range) %
Latissimus Dorsi	6.5 (0.0–36.4)
Teres Major	4.2
Lower Trapezius	7.9 (7.1–8.3)
Middle Trapezius	6.3 (4.5–7.7)
Pectoralis Major	14.1 (10.0–36.4)
Combined (LD & TM)	9.5 (6.7–11.5)

The LD TT demonstrated a weighted mean failure rate of 6.5%, with a broad range spanning from 0.0% to 36.4%. The TM TT had the lowest reported failure rate at 4.2%, though this value reflects data from a single study,^37^ limiting its generalizability.

The LT TT exhibited a mean failure rate of 7.9%, with a narrow range of 7.1% to 8.3%, indicating consistent outcomes across studies. Similarly, the MT TT showed a comparable failure rate of 6.3%, with a range of 4.5% to 7.7%; however, this reflects a limited number of studies with relatively small sample sizes, and results should therefore be interpreted with caution.

PM transfers had the highest weighted mean failure rate among all tendon types, at 14.1%, with variability ranging from 10.0% to 36.4%. The combined LD & TM TTs yielded a failure rate of 9.5% (range 6.7–11.5%) across three studies, representing intermediate outcomes compared to individual TTs. However, evidence remains preliminary due to the small volume of available data (Table 4).

### Complications

The analysis of complications across the 30 studies revealed a variety of postoperative issues associated with TT surgeries for IRCTs. Table 5 summarizes the most commonly reported post-operative complications categorized by tendon type.

**Table 5.** Most commonly reported post-operative complications following tendon transfers for irreparable rotator cuff tears.

Complications	Latissimus Dorsi	Teres Major	Lower Trapezius	Middle Trapezius	Pectoralis Major	Combined Transfer	Total cases
Infections	10 (1.7%)	0 (0.0%)	0 (0.0%)	2 (4.2%)	3 (2.0%)	2 (2.7%)	17
Hematoma	14 (2.4%)	0 (0.0%)	0 (0.0%)	0 (0.0%)	3 (2.0%)	0 (0.0%)	17
*Rotator Cuff Arthropathy	20 (3.4%)	1 (4.2%)	2 (1.8%)	0 (0.0%)	3 (2.0%)	5 (6.6%)	31

*Hamada Grade 3/4 or progressed to severe as stated by the authors.

Seventeen cases of infection were noted across 12 studies.^19,21–23,33,35,36,38,39,43–45^ Within the LD transfer group, which included 585 shoulders, 10 infections were reported, corresponding to approximately 1.7% of LD cases. Other tendon types demonstrated varying infection rates, including 4.2% for MT, 2.0% for PM, and 2.7% for combined transfers. Hematoma formation was also observed in 17 cases across 8 studies.^23,25,29,33,35,36,41,45^ This complication occurred in 2.4% of shoulders that underwent LD transfers and 2.0% of shoulders receiving PM transfers. No hematomas were reported in the TM, MT, LT, or combined transfer groups. Rotator cuff arthropathy, defined as Hamada Grade 3 or 4 arthropathy or progression to severe rotator cuff osteoarthritis, was another frequently reported complication, documented in 11 studies.^18,20,22,25–27,30,34,37,41,43^ This complication was most prevalent in the combined transfer group, affecting 6.6% of patients, compared to 3.4% in the LD group, 4.2% in the TM group, 2.0% in the PM group, and 1.8% in the LT group. Notably, no cases were reported in the MT transfer cohort (Table 5).

In addition to the commonly reported complications, several less frequent but noteworthy complications were documented. Axillary nerve palsy was reported in four cases across three studies.^19,25,36^ One case was reported in the combined transfer group while three cases were reported in the LD transfer cohort. Radial nerve palsy and brachial plexus injury were noted in two studies^25,35^ and one study,^29^ respectively, all in the LD transfer group. These were mostly attributed to excessive tension or traction during tendon mobilization or passage, particularly when performed through limited exposure. Axillary wound dehiscence was reported in two patients across two different studies^21,35^ and frozen shoulder was noted in two cases following LD transfers.^25^ One case of a deep vein thrombosis was reported in the axillary vein following PM TT.^44^

## Discussion

This systematic review provides a comprehensive synthesis of the available literature on TTs for IRCTs. The analysis included 30 studies, comprising 980 patients and 994 shoulder repairs, with an overall mean age of 58.9 years and a mean follow-up of 44.7 months. The findings highlight key differences in PROMs, surgical outcomes, and indications for the various tendon types utilized.

The LD tendon is historically the oldest studied tendon in clinical practice, particularly in the management of posterosuperior IRCTs. The findings in this review align with previous studies,^46,47^ demonstrating similar clinical success at improving PROMs.^48^ This was suggested due to the biomechanical suitability of the LD tendon at restoring shoulder function through balanced force coupling.^49,50^ Successful TT outcomes depend not only on tendon selection but also on restoration of balanced force coupling, the presence of a functional subscapularis, and preserved forward elevation greater than 90°, as emphasized in biomechanical studies.^49,50^

PM TTs were solely used to treat anterosuperior IRCTs. A systematic review by Luo et al. (2022)^51^ reported failure rates similar to those observed in this study, raising important clinical concerns about the underlying causes of PM TT failures. Within this review, most PM TT failures were attributed to avulsion at the osteotendinous junction (tendon-bone interface),^40,41,43,44^ or unspecified ruptures.^42,45^ This highlights the critical need for secure osseous fixation to facilitate adequate bony ingrowth and proper healing of the transferred tendon at its new insertion site.^40,44^ The relatively high failure rates associated with PM TTs may also stem from the lack of standardized surgical techniques across studies. Variations such as the use of different clavicular heads (sternal, clavicular, or both) and whether the TT was routed under or over the conjoint tendon can significantly influence outcomes.^40,44,50^ Furthermore, the biomechanical line of pull of the PM transfer relative to the subscapularis muscle is a key consideration. If the PM is not routed beneath the conjoint tendon, it may exert an anterior pull on the humerus, leading to excessive stress at the tendon-bone interface. This stress, caused by a tractional rather than rotational force, can compromise tendon healing.^40^ Given the PM's substantial traction force, these surgical variations can alter the biomechanical tension on the transfer, impacting its success.^40^ PM TTs were often performed as salvage procedures, frequently in the context of additional injuries, such as posterosuperior IRCTs, which may have further contributed to suboptimal outcomes.^50^ The high failure rate of PM TTs should prompt caution in their use for anterosuperior IRCTs, particularly in younger patients or those with high functional demands. Surgeons should be aware of the potential for postoperative complications, including nerve injuries and persistent pain, which were reported across several studies.

Attention was also drawn to TM and LT transfers, which, although less commonly studied, showed promise for specific clinical indications. These transfers may provide notable biomechanical advantages due to their vector of pull closely replicating that of the infraspinatus muscle, a finding corroborated by studies such as those by Steenbrink and others.^53–55^

The inclusion of studies utilizing LT tendons supports the growing interest in this option, particularly for patients with posterosuperior IRCTs.^56^ Biomechanical analyses by Hartzler et al. (2012) also supported LT TTs, as their line of pull and external rotation moments closely resemble those of the infraspinatus.^49^ However, slightly higher failure rates (7.9%) were noted in the studies involving LT TTs, primarily at the graft attachment sites on the rotator cuff footprints.^57–59^ These failures may be attributed to the use of bridging allografts, which are often employed in LT transfers but may not heal as reliably as autografts. Additionally, excessive tension applied to achieve sufficient excursion can lead to progressive deformation of the interpositional graft over time.^39^ Despite these challenges, LT transfers showed promising functional outcome scores and warrant further investigation due to their better line of pull and potential applicability in more complex patient populations, such as those with brachial plexus injuries.^56^ Continued research could help refine techniques and improve outcomes in these challenging cases.

Research into combined LD + TM transfers is still in its infancy. The reported failure rate of 9.5%, while modest, is still greater than each TT taken in isolation for posterosuperior IRCTs. This indicates that a combined approach may not currently yield the improved outcomes suggested by Adam et al. (2021).^50^ One possible reason for the slightly higher failure rates could be that combined TTs face more complex demands in terms of tendon excursion to reach the footprint of the posterosuperior rotator cuff, potentially leading to excessive tension on the TT.^52^ However, this finding could also be attributed to the small sample sizes and limited statistical power of the studies reviewed, underscoring the need for further research. Another concern with the combined LD + TM transfer is that both tendons are shoulder adductors. Taking both tendons leaves only the PM tendon as the only adductor of the shoulder, thus significantly weakening shoulder adduction.

The MT TT, while less commonly studied, offers a unique approach for addressing IISSTs. MT transfers provide the benefit of a biomechanical line of pull that closely mimics the supraspinatus, contributing to improved shoulder elevation.^38,39^ Recent studies have demonstrated moderate success in pain relief and functional outcomes, with pooled improvements in Constant-Murley and ASES scores.^38,39^ However, the slightly elevated failure rates observed, particularly at the tendon-bone interface, suggest that further refinement of fixation techniques may be necessary to optimize healing.^38,39^ As a relatively new procedure, MT transfers hold promise but require additional research to confirm their efficacy and expand their indications beyond isolated supraspinatus tears.

Overall, the choice of tendon for transfer appears to depend on several factors, including the location and extent of the RCT, patient age, and comorbidities. LD transfers remain the most studied and common option for posterosuperior IRCTs, offering reliable improvements in pain and function and relatively low failure rates. PM transfers provide an alternative for anterosuperior IRCTs, particularly when the subscapularis is involved. The utility of LT and MT transfers may be more context-specific, with LT transfers favored for posterosuperior tears and MT transfers for isolated supraspinatus tears. The limited evidence for TM transfers suggests potential but requires further investigation to establish its role in clinical practice.

Common challenges among these treatments for IRCTs include TT tears, infections, suture anchor complications, and the need for reoperation, indicating a complex and high-risk profile for these procedures.^60–63^ Patients with prior rotator cuff repair have higher rates of revision surgeries and complications (11% and 18.2%, respectively) than those who undergo primary RTSA (3% and 2.9%, respectively).^64^

Overall, the complications identified in this analysis underscore the need for careful patient selection and thorough preoperative assessment. Differences in complications between procedures highlight the necessity for clear patient education and effective counselling so patients can make informed decisions that align with their expectations. Proper dissection planes and minimizing intraoperative traction are essential strategies to reduce the risk of nerve injury.

The clinical significance of PROM improvements hinges on understanding the minimum clinically important difference (MCID).^65^ The MCIDs for the CMS, ASES, UCLA, and VAS pain scale are considered to be an increase of 10.4 points,^66^ 15.2–27.1 points,^67,68^ 3.5 points,^67^ and a reduction of 1.4 points,^68^ respectively. The tendon-specific PROM improvements exceeded the MCID thresholds in most cases, suggesting that the majority of patients with IRCTs experienced meaningful functional recovery and pain management post-operatively. However, LT transfers resulted in an ASES improvement of +26.9, which, while significant, did not reach the upper limit of the MCID range for this outcome measure.

The overall quality of included studies, as assessed by MINORS criteria, revealed significant variability. Non-comparative studies were predominantly of moderate quality, with three rated as poor. Comparative studies demonstrated higher methodological rigor, though none achieved the highest quality rating. Common reasons included the lack of unbiased endpoints, inadequate sample size calculations, and inconsistent reporting of outcome measures. This variability underscores the need for standardized, high-quality studies to better evaluate TT procedures.

Several limitations of this review must be acknowledged. First, the majority of included studies were retrospective in nature, introducing the potential for selection bias and limiting the generalizability of the findings. Second, significant heterogeneity existed across studies in terms of surgical techniques (e.g. onlay vs inlay fixation for LD transfers, open vs arthroscopic-assisted vs fully arthroscopic approaches, and variations in routing for PM transfers), failure definitions, and indications, complicating direct comparisons between different TT types.^35,36,56,58^ Additionally, the type and location of tendon fixation, such as anchoring at the supraspinatus versus infraspinatus footprint, may influence both biomechanical effectiveness and complication rates, including failure.^69^ Postoperative rehabilitation protocols were also variably described, with differences in immobilization duration, timing of passive versus active range of motion, and progression to strengthening. These important surgical variables were inconsistently reported across studies, limiting subgroup analyses. This variability likely influenced outcomes but was inconsistently reported, precluding meaningful subgroup analyses. Preoperative assessment factors, such as forward elevation above 90°, external rotation lag, and reparability of the subscapularis, were variably considered across studies, further limiting the ability to evaluate prognostic indicators. Functional recovery outcomes, including return to work and activities of daily living, as well as surgeon experience and learning curve effects, were also inconsistently reported, restricting insights into real-world effectiveness. The limited number of high-quality comparative studies and variability in follow-up durations similarly hinder definitive conclusions regarding the long-term efficacy of specific transfers. Furthermore, the underrepresentation of certain tendons, such as the TM and combined TM + LD transfers, restricts broader understanding of their clinical applicability. Finally, although PROMs provide valuable insights into perceived improvements, objective assessments such as imaging-confirmed healing and muscle strength testing were inconsistently reported, potentially affecting the interpretation of clinical success.

This is considered the first systematic review to highlight clinical outcomes and tendon failure rates of TTs for IRCTs. Consolidating evidence from multiple sources, this review facilitates evidence-based decision making, patient education, and highlights areas for future research to further treatment strategies for IRCTs. Future research should focus on conducting high-quality prospective, randomized controlled trials to compare the efficacy and safety of different TTs. Given that IRCTs comprise only a small proportion of surgically treated RCTs, adequately powered prospective trials may only be feasible through multicenter collaboration or at high-volume shoulder centers. Such studies are needed to directly compare LD (or LD + TM) versus LT transfers for posterosuperior IRCTs, and LD (or LD + TM) versus PM transfers for anterosuperior IRCTs. In particular, further investigation into the use of the TM and LT TTs is warranted, as these options have shown potential but remain underrepresented in the literature. Standardization of outcome measures, such as the use of the CMS, ASES, and VAS, will facilitate more robust comparisons between studies and assist in determining the impact of various demographic factors on PROMs. Additionally, future studies should evaluate the impact of rehabilitation protocols on postoperative outcomes, as variations in postoperative care may contribute to differences in reported success rates. Long-term follow-up is essential to assess the durability of these repairs, particularly given the potential for progressive joint degeneration and TT failure. Future systematic reviews focusing separately on posterosuperior and anterosuperior IRCTs may help refine surgical indications and tendon choice more precisely.

## Conclusion

This systematic review underscores the effectiveness of TTs in improving pain and function for patients with IRCTs, while highlighting variations in outcomes based on tendon type and surgical indications. The varying failure rates and complication profiles necessitate careful consideration of TT selection tailored to individual patient needs and the type of IRCT. While the LD remains the most commonly used tendon for posterosuperior IRCTS, emerging evidence for alternative tendon types such as TM, LT, or combined LD & TM suggests expanding options for tailored surgical management. PM transfers provide an alternative for anterosuperior IRCTs, particularly when the subscapularis is involved while MT transfers have shown some promise in the management of isolated supraspinatus tears. Future high-quality, standardized research is essential to refine surgical techniques, establish evidence-based guidelines, and optimize patient outcomes in this challenging cohort.

## Supplemental Material

sj-docx-1-sel-10.1177_17585732251368884 - Supplemental material for Evaluating tendon transfers in irreparable rotator cuff tears: A systematic review of clinical outcomes and failure rates Supplemental material, sj-docx-1-sel-10.1177_17585732251368884 for Evaluating tendon transfers in irreparable rotator cuff tears: A systematic review of clinical outcomes and failure rates by Marc Daniel Bouchard, Nikhil Aman Patel, Carl Keogh, Danielle Dagher, Brooke Mackinnon, Sophia Argyropoulos, David Slawaska-Eng, Moin Khan and Bashar Alolabi in Shoulder & Elbow

sj-docx-2-sel-10.1177_17585732251368884 - Supplemental material for Evaluating tendon transfers in irreparable rotator cuff tears: A systematic review of clinical outcomes and failure rates Supplemental material, sj-docx-2-sel-10.1177_17585732251368884 for Evaluating tendon transfers in irreparable rotator cuff tears: A systematic review of clinical outcomes and failure rates by Marc Daniel Bouchard, Nikhil Aman Patel, Carl Keogh, Danielle Dagher, Brooke Mackinnon, Sophia Argyropoulos, David Slawaska-Eng, Moin Khan and Bashar Alolabi in Shoulder & Elbow
