# Supplementary material for: Evaluating tendon transfers in irreparable rotator cuff tears: A systematic review of clinical outcomes and failure rates
Source: Shoulder Elbow. 2025 Aug 14:17585732251368884. Online ahead of print. doi: 10.1177/17585732251368884 (PMC12354401; doi:10.1177/17585732251368884)
Supplement: sj-docx-1-sel-10.1177_17585732251368884 - Supplemental material for Evaluating tendon transfers in irreparable rotator cuff tears: A systematic review of clinical outcomes and failure rates [file sj-docx-1-sel-10.1177_17585732251368884.docx]

**Supplementary Table i).** MeSH terms and Boolean operators used to develop search strategy from Embase, Emcare and OVID Medline databases.

**Database: Embase <1974 to 2024 August 12>**
**Search Strategy:**
**1**  rotator cuff injury/ or rotator cuff rupture/ or rotator cuff tear arthropathy/ (14435)
**2**  (rotator cuff adj3 (tear* or injur* or rupture)).ti,ab,kw,kf. (11000)
**3**  1 or 2 (16854)
**4**  irreparable.mp. (4138)
**5**  3 and 4 (1365)
**6**  tendon transfer/ (4792)
**7**  tendon transfer*.ti,ab,kw,kf. (3919)
**8**  tendon/su [Surgery] (1223)
**9**  "lattisimus dorsi tendon transfer".mp. (0)
**10**  lat tendon transfer.mp. (0)
**11**  trapezius tendon transfer.mp. (54)
**12**  rotator cuff tendon transfer.mp. (1)
**13**  or/6-12 (7057)
**14**  5 and 13 (356)

**Database: Ovid Emcare <1995 to 2024 Week 32>**
**Search Strategy:**
**1**  rotator cuff injury/ or rotator cuff rupture/ or rotator cuff tear arthropathy/ (4584)
**2**  (rotator cuff adj3 (tear* or injur* or rupture)).ti,ab,kw,kf. (5431)
**3**  1 or 2 (7178)
**4**  irreparable.mp. (1390)
**5**  3 and 4 (647)
**6**  tendon transfer/ (1259)
**7**  tendon transfer*.ti,ab,kw,kf. (1768)
**8**  [tendon/su [Surgery]] (0)
**9**  "lattisimus dorsi tendon transfer".mp. (0)
**10**  lat tendon transfer.mp. (0)
**11**  trapezius tendon transfer.mp. (23)
**12**  rotator cuff tendon transfer.mp. (1)
**13**  or/6-12 (2101)
**14**  5 and 13 (159)

**Database: Ovid MEDLINE(R) ALL <1946 to August 12, 2024>**
**Search Strategy:**
**1**  Rotator Cuff Injuries/ (8292)
**2**  (rotator cuff adj3 (tear* or injur*)).ti,ab,kw,kf. (8920)
**3**  1 or 2 (12084)
**4**  irreparable.mp. (3329)
**5**  3 and 4 (1125)
**6**  Tendon Transfer/ (4616)
**7**  tendon transfer*.ti,ab,kw,kf. (3347)
**8**  Tendons/su [Surgery] (7805)
**9**  "lattisimus dorsi tendon transfer".ti,ab,kw,kf. (0)
**10**  lat tendon transfer.mp. (0)
**11**  trapezius tendon transfer.mp. (40)
**12**  rotator cuff tendon transfer.mp. (1)
**13**  or/6-12 (13160)
**14**  5 and 13 (347)
