# Supplementary material for: Evaluating tendon transfers in irreparable rotator cuff tears: A systematic review of clinical outcomes and failure rates
Source: Shoulder Elbow. 2025 Aug 14:17585732251368884. Online ahead of print. doi: 10.1177/17585732251368884 (PMC12354401; doi:10.1177/17585732251368884)
Supplement: sj-docx-2-sel-10.1177_17585732251368884 - Supplemental material for Evaluating tendon transfers in irreparable rotator cuff tears: A systematic review of clinical outcomes and failure rates [file sj-docx-2-sel-10.1177_17585732251368884.docx]

**Supplementary Table ii).** MINORS score calculations quality assessment of all included papers in review.

|  | 1. Clearly stated aim | 2. Inclusion of consecutive patients | 3. Prospective data collection | 4. Endpoints appropriate to the aim of the study | 5. Unbiased assessment of the study endpoint | 6. Follow-up period appropriate to the aim of the study | 7. Loss to follow up less than 5% | 8. Prospective calculation of the study size | 9. Adequate control group | 10. Contemporary groups | 11. Baseline equivalence of groups | 12. Adequate statistical analysis | **Total Minors Score** | **Maximum possible score** |
| --- | --- | --- | --- | --- | --- | --- | --- | --- | --- | --- | --- | --- | --- | --- |
| Gerhardt et al. (2010)^18^ | 2 | 2 | 1 | 2 | 0 | 2 | 0 | 0 |  |  |  |  | **9** | **16** |
| **Baek et al. (2022)^19^** | 2 | 2 | 1 | 2 | 1 | 2 | 0 | 0 | 2 | 1 | 2 | 1 | **16** | **24** |
| Boileau et al. (2018)^20^ | 2 | 2 | 1 | 2 | 0 | 2 | 1 | 0 |  |  |  |  | **10** | **16** |
| Elhassan et al. (2020)^21^ | 2 | 2 | 1 | 2 | 0 | 1 | 0 | 0 |  |  |  |  | **8** | **16** |
| Suh et al. (2019)^22^ | 2 | 2 | 1 | 2 | 0 | 2 | 0 | 0 |  |  |  |  | **9** | **16** |
| Grimberg et al. (2015)^23^ | 2 | 2 | 1 | 2 | 1 | 2 | 2 | 0 |  |  |  |  | **12** | **16** |
| Warner et al. (2001)^24^ | 2 | 2 | 1 | 2 | 0 | 2 | 0 | 0 |  |  |  |  | **9** | **16** |
| El-Azab et al. (2015)^25^ | 2 | 2 | 1 | 2 | 1 | 2 | 0 | 1 |  |  |  |  | **11** | **16** |
| Aoki et al. (1996)^26^ | 0 | 2 | 2 | 1 | 0 | 2 | 2 | 0 |  |  |  |  | **9** | **16** |
| Nové-Josserand et al. (2008)^26^ | 0 | 2 | 0 | 1 | 0 | 2 | 1 | 0 |  |  |  |  | **6** | **16** |
| Clavert et al. (2020)^28^ | 2 | 2 | 1 | 2 | 0 | 2 | 2 | 0 |  |  |  |  | **11** | **16** |
| Hanson et al. (2023)^12^ | 2 | 2 | 2 | 2 | 0 | 2 | 2 | 0 | 2 | 2 | 1 | 2 | **19** | **24** |
| Valenti et al. (2019)^29^ | 2 | 2 | 2 | 2 | 0 | 2 | 2 | 0 | 2 | 2 | 0 | 2 | **18** | **24** |
| Ersen et al. (2014)^30^ | 2 | 2 | 1 | 2 | 0 | 2 | 2 | 0 |  |  |  |  | **11** | **16** |
| Iannotti et al. (2006)^31^ | 2 | 2 | 1 | 2 | 2 | 2 | 0 | 0 |  |  |  |  | **11** | **16** |
| Paribelli et al. (2015)^32^ | 2 | 2 | 2 | 2 | 0 | 2 | 0 | 2 | 2 | 2 | 2 | 2 | **20** | **24** |
| Zafra et al. (2009)^33^ | 1 | 0 | 1 | 0 | 0 | 2 | 0 | 0 |  |  |  |  | **4** | **16** |
| **Baek et al. (2022)^34^** | 2 | 2 | 1 | 2 | 1 | 2 | 0 | 0 |  |  |  |  | **10** | **16** |
| Kany et al. (2024)^35^ | 2 | 2 | 1 | 2 | 0 | 2 | 1 | 2 | 2 | 2 | 2 | 2 | **20** | **24** |
| Kany et al. (2023)^36^ | 2 | 2 | 1 | 2 | 0 | 2 | 2 | 2 | 2 | 2 | 2 | 2 | **21** | **24** |
| Celli et al (2023)^37^ | 2 | 2 | 1 | 2 | 0 | 2 | 0 | 0 |  |  |  |  | **9** | **16** |
| Kim et al. (2024)^38^ | 2 | 2 | 1 | 2 | 2 | 2 | 0 | 0 | 2 | 2 | 2 | 2 | **19** | **24** |
| Marigi et al. (2023)^13^ | 2 | 2 | 1 | 2 | 1 | 2 | 2 | 0 | 2 | 2 | 1 | 2 | **19** | **24** |
| **Baek et al. (2023)^39^** | 2 | 2 | 1 | 2 | 2 | 2 | 2 | 0 |  |  |  |  | **13** | **16** |
| Lederer et. al (2011)^40^ | 2 | 2 | 0 | 2 | 1 | 2 | 1 | 0 | 2 | 2 | 2 | 2 | **18** | **24** |
| Gavriilidis et al. (2009)^41^ | 2 | 1 | 1 | 2 | 1 | 2 | 2 | 0 |  |  |  |  | **10** | **16** |
| Elhassan et al. (2008)^42^ | 2 | 2 | 1 | 2 | 1 | 2 | 1 | 0 | 2 | 2 | 1 | 2 | **18** | **24** |
| Ernstbrunner et al. (2019)^43^ | 2 | 2 | 1 | 2 | 1 | 2 | 1 | 0 |  |  |  |  | **11** | **16** |
| Jost et al. (2003)^44^ | 2 | 2 | 1 | 2 | 2 | 2 | 2 | 0 | 1 | 2 | 2 | 2 | **20** | **24** |
| Valenti et al. (2014)^45^ | 2 | 1 | 2 | 2 | 1 | 2 | 2 | 0 | 2 | 2 | 1 | 2 | **19** | **24** |

**Legend (Total MINORS Score)** poor quality; moderate quality; good quality
